# Supplementary material for: Negative Influence of Aging on Differentiation and Proliferation of CD8+ T-Cells in Dogs
Source: Vet Sci. 2023 Aug 25;10(9):541. doi: 10.3390/vetsci10090541 (PMC10534501; doi:10.3390/vetsci10090541)
Supplement: Supplementary file 1 [file vetsci-10-00541-s001.zip › vetsci-2463760-supplementary.pdf]

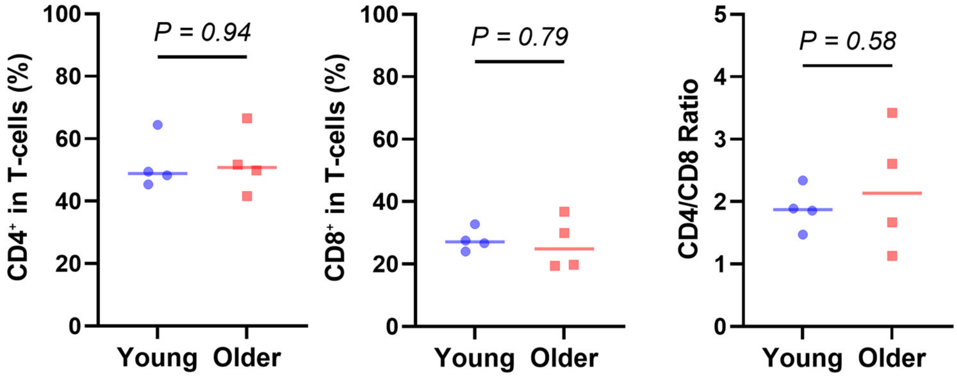

Figure S1. Percentage and ratio of $CD4^+$ or $CD8^+$ T-cells in peripheral blood of young and older dogs. Each plot represents individual data. n = 4 for each group.

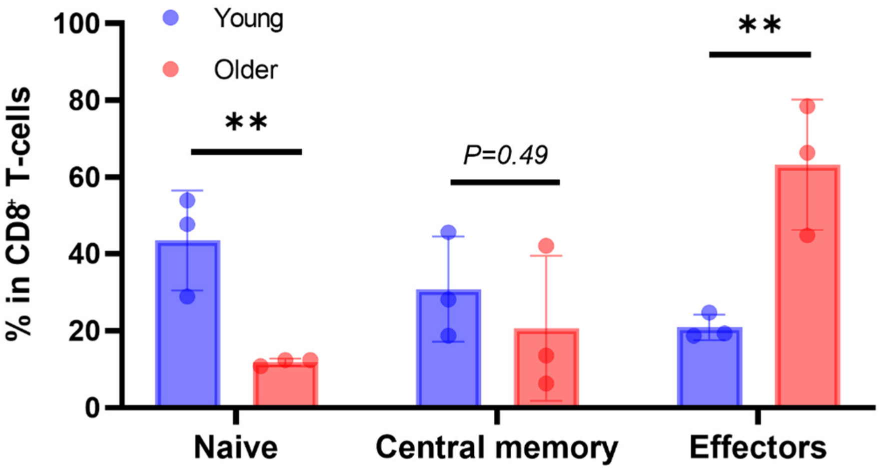

Figure S2. Percentage of $CD8^+$ T-cell subsets at day 8 post-stimulation. Each plot represents individual data. ** $p < 0.01$. n = 3 for each group.

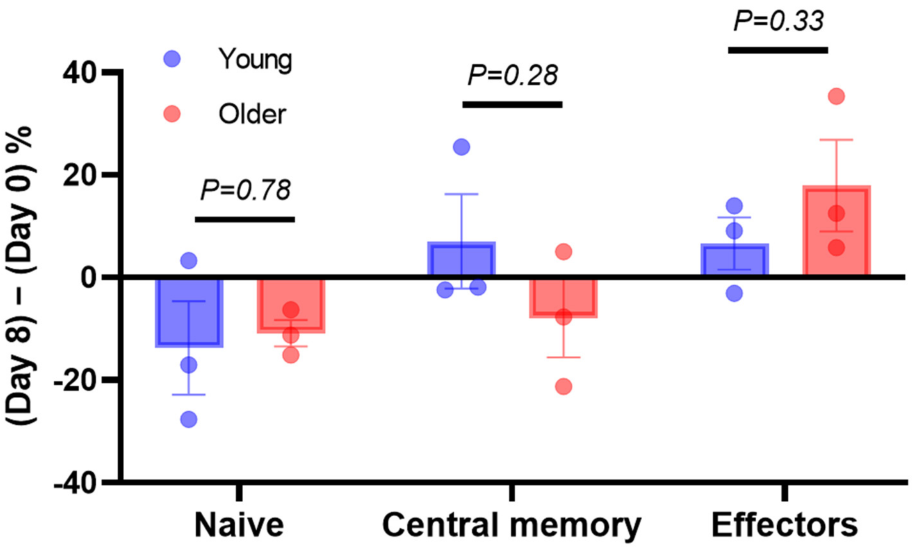

Figure S3. Percentage changes in subsets in CD8+ T-cells from day 0 to day 8 post-stimulation. Each plot represents individual data. n = 3 for each group.

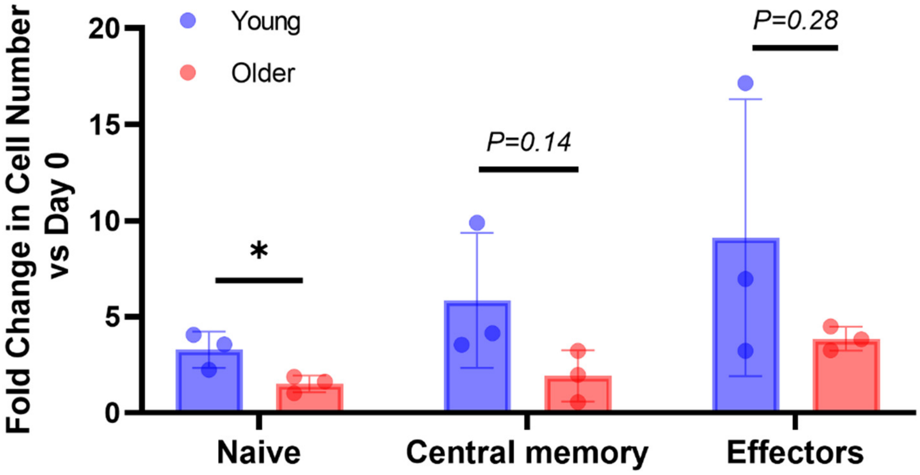

Figure S4. Fold changes in cell numbers of each CD8+ T-cell subsets on day 8 post-stimulation compared with pre-stimulation. Each plot represents individual data. *$p < 0.05$. n = 3 for each group.
